# Triple Threat: Elucidating Evolution Under Antibiotic, Metal, and Phage Pressure in *Escherichia coli*

**DOI:** 10.1002/ece3.74239

**Published:** 2026-08-27

**Authors:** Lindsey W. McGee, Noah K. Radtke, Gretchen S. Taylor, Joseph L. Graves, Liesl Jeffers‐Francis

**Affiliations:** ^1^ Biology Department Earlham College Richmond Indiana USA; ^2^ North Carolina A&T State University Greensboro North Carolina USA

**Keywords:** bacteriophage, *E. coli*, epistasis, pleiotropy, resistance, trade‐offs

## Abstract

Antibiotic resistance is a growing challenge in the treatment of bacterial infections, prompting interest in alternative therapies. Understanding how bacterial populations evolve under different selective pressures is important for predicting resistance pathways. This study aimed to investigate the evolutionary responses of *E. coli* populations to different antimicrobial environments and assess how these conditions influence resistance to phage infection, ampicillin, and gallium nitrate. Populations of EC‐WT and EC‐Phage‐Resistant *E. coli* were experimentally evolved for 10 days under different selection conditions: ampicillin, gallium nitrate, and a combination of ampicillin and gallium nitrate. Following the evolution period, phage resistance was assessed using spot assays. Growth fitness in the presence of ampicillin and gallium nitrate was measured using assays conducted in 96‐well plates. Whole‐genome sequencing was performed to identify mutations associated with adaptive responses. Several evolved populations developed resistance across multiple treatments regardless of the initial selection condition. Notably, all phage‐resistant mutants exhibited triple resistance to phage infection, ampicillin, and gallium nitrate. Sequencing analyses revealed multiple mutations associated with increased growth fitness under ampicillin and gallium nitrate exposure. These findings demonstrate that *E. coli* populations can rapidly evolve multidrug resistance under diverse selection pressures. In some populations, epistatic interactions between preexisting phage‐resistance mutations and newly acquired mutations limited growth‐rate fitness, suggesting that such interactions may constrain certain evolutionary pathways of resistance. Examining phage therapy through an evolutionary framework can help identify potential resistance pathways and inform the development of more effective strategies for treating multidrug‐resistant bacterial infections.

## Graphical abstract

Antibiotic resistance threatens treatment of bacterial infections, increasing research into alternative therapies, in particular whether new resistances will arise to these novel treatments. We evolved bacteria under phage, antibiotic, and heavy metal exposure. We predicted that evolutionary trade‐offs would limit resistance arising, but instead found many instances of multi‐treatment resistance.

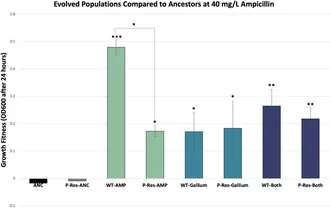

## 1 Background and Objectives

The invention of antibiotics stands as one of the most pivotal milestones in human history, playing a vital role in preserving lives (Andersson and Hughes 2010). Antimicrobial resistance has emerged as a critical global health threat, undermining the efficacy of existing antimicrobial agents, such as antibiotics, and complicating the treatment of many bacterial infectious diseases. The evolution of bacterial resistance has resulted in multidrug‐resistant pathogens, including the clinically important ESKAPE pathogens (Andersson 2003; De Oliveira et al. 2020; Miller and Arias 2024). The World Health Organization (WHO) has deemed AMR as one of the top ten global public health threats, with bacterial resistance directly responsible for an estimated 1.27 million deaths worldwide in 2019, contributing to a total of 4.95 million deaths associated with resistant infections (WHO 2026). Without effective interventions, antimicrobial‐resistant pathogens are projected to cause 10 million deaths annually by 2050 (WHO 2026). The overuse and misuse of antimicrobials in human medicine, veterinary applications, and agriculture have accelerated the emergence of multidrug‐resistant (MDR) bacterial strains, necessitating the development of alternative therapeutic approaches (WHO 2026).

Phage therapy, which utilizes bacteriophages—viruses that selectively infect and lyse bacterial cells—is a potential alternative approach to antibiotic treatment (Kortright et al. 2019; Loc‐Carrillo and Abedon 2011). While bacteriophages have been historically used in antimicrobial treatments, renewed interest in their application has emerged due to their specificity, ability to coevolve with resistant bacteria, and potential to target MDR pathogens (Lin et al. 2022). However, bacteria can also evolve resistance to phages through mechanisms such as receptor modification or loss, secretion of anti‐adhesion substances, blockage of phage DNA injection, and inhibition of phage replication and release (Hyman and Abedon 2010; Principi et al. 2019; Seed et al. 2012), highlighting the need for complementary therapeutic strategies.

One approach to mitigate this problem is combinatorial approaches with phage therapy and other agents that affect bacterial cell growth and viability (Tagliaferri et al. 2019). For instance, Chaudhry et al. (2017) observed a phage‐antibiotic combination synergistically enhancing biofilm reduction in *P. aeruginosa* , with its effectiveness depending on the antibiotic type, dosage, and timing of administration. In addition, studies conducted by Ryan et al. (2012) and Coulter et al. (2014) have shown that combining phage with antibiotics greatly enhanced the eradication of biofilms in *Escherichia coli*, reduced the needed antibiotic dosage for treatment, and diminished the number of resistant bacteria emerging. However, phage‐antibiotic combinatory approaches can cause unexpected and detrimental outcomes as well. While there is copious research done that supports the notion of the combinatory strategy being beneficial, some have found negative interference, in which antibiotic treatment reduces bacterial metabolic activity or replication to a level that limits phage adsorption, replication, or lytic activity, thereby decreasing the overall efficiency of the combination therapy (Tagliaferri et al. 2019). Hence, elucidating the overall effect of phage‐antibiotic combination treatment is necessary to further evaluate its efficacy and safety.

Another combinatorial approach that has been examined is the interaction between bacteriophages and heavy metals. Heavy metal exposure is a factor known to impact bacterial evolution and resistance development. Certain metals, such as gallium, have demonstrated antimicrobial properties by disrupting bacterial iron metabolism and inhibiting biofilm formation (Kaneko et al. 2007). Gallium's mechanism of action mimics iron, an essential nutrient for bacterial growth, but fails to participate in vital metabolic processes, leading to bacterial growth inhibition (Graves et al. 2019). Additionally, studies have shown that bacteria can rapidly evolve resistance to gallium, sometimes leading to trade‐offs in fitness between competing traits due to pleiotropy (Graves et al. 2019; Choi et al. 2020). Pleiotropy describes a situation in which a single locus or mutation in the gene influences more than one phenotypic trait (Stearns 2010). Furthermore, exposure to heavy metals can induce mutations and stress responses in bacterial populations, which may contribute to cross‐resistance or trade‐offs in fitness under different selective pressures (Hobman and Crossman 2015; Lemire et al. 2013).

Understanding the evolutionary landscapes of how bacterial adaptation is guided by the concepts of fitness valleys and evolutionary traps can be crucial to being able to suppress bacterial population growth and the evolution of resistance. Evolutionary landscapes are often shown as a three‐dimensional surface with fitness peaks and valleys, illustrating how fitness varies across potential evolutionary trajectories (Steinberg and Ostermeier 2016a; Wright 1932). Fitness valleys refer to a region in the fitness landscape where organisms have reduced fitness, and peaks represent high‐fitness conditions. Organisms occupying a fitness valley experience reduced fitness and often require multiple mutations before reaching a higher fitness adaptive peak, particularly following changes in the environment (Wright 1932). An evolutionary trap occurs when environmental conditions and/or epistatic interactions constrain these adaptive trajectories, preventing populations from evolving toward higher fitness, resistant genotypes (Steinberg and Ostermeier 2016b). In the context of antimicrobial treatment against MDR pathogens, strategically combining phage therapy, antibiotics, and heavy metals may help contain bacterial populations at a lower fitness level, limiting their ability to evolve resistance. In addition, we hope to gain insights into how we can predict evolutionary pathways, which would also be beneficial in terms of devising novel therapeutic strategies.

In this context, our study aims to investigate potential evolutionary trade‐offs and adaptive constraints in *Escherichia coli* populations subjected to different selective pressures, including phage infection, antibiotic treatment, and metal exposure. Specifically, we sought to determine whether resistance to one stressor confers increased susceptibility to another. To address this question, we exposed both wild‐type and phage‐resistant *E. coli* strains to the following conditions: antibiotic (Ampicillin), heavy metal (Gallium nitrate), and a combination of antibiotic and heavy metal. Using experimental evolution, we assessed how ecological complexity influences the genetic architecture underlying adaptation and the evolution of multi‐treatment resistance. By analyzing the evolutionary outcomes of *E. coli* under these treatment conditions, we aim to elucidate whether selective pressures drive or overcome fitness trade‐offs that could inform novel therapeutic strategies for MDR bacterial infections.

## 2 Methodology

### 2.1 Strains and Growth Conditions

We grew bacteria in lysogeny broth (LB) with 10 g tryptone, 5 g yeast extract, and 10 g/L NaCl. LB plates included 15 g/L agar, or 7.5 g/L for top agar. For liquid cultures, overnight incubation was performed at 200 rpm shaking at 37°C. For agar cultures, overnight incubation was performed in a cabinet incubator at 37°C. Growth conditions and media preparation followed standard bacteriological methods (Bertani 1951; Sambrook and Russell 2001).

Previously isolated phage‐resistant *E. coli* populations (McGee et al. 2023) fixed mutations that affect lipopolysaccharide (LPS) structure and/or synthesis when challenged with Microviridae strain WA11. The bacteriophage used for phenotypic assays was Microviridae phage WA11, originally isolated and described by Rokyta et al. (2009). Phage stocks were propagated on the ancestral *E. coli* C host strain and stored following the procedures described by Rokyta et al. (2009). The phage‐resistant *E. coli* population used for this experiment fixed the point mutation H160L (CAC → CTC) located in the rfaP gene. The rfaP protein is involved in the pathway for LPS core biosynthesis as part of the bacterial outer membrane (Bateman et al. 2021). Mutations in the LPS structure and synthesis may have disabled the use of LPS as a receptor for absorption mechanisms, thereby preventing phage entry to the host cell.

### 2.2 Experimental Evolution

To gain a better understanding of the evolutionary pathways of *E. coli* , both wild‐type *E. coli* strain *C* (EC‐WT) and phage resistant *E. coli* (mutant rfaP; EC‐P‐Res) were evolved over 10 days using procedures previously described in Graves et al. (2019). In our experiments, we evolved 24 independent populations of *E. coli* to different selection conditions: ampicillin, gallium nitrate, and ampicillin/gallium nitrate. For each genotype, independent replicate populations were founded from individual colonies isolated from the ancestral stock by serial dilution plating. Each replicate population was propagated in separate culture flasks containing LB medium supplemented with the appropriate selective agent(s). Cultures were incubated for 24 h at 37°C with shaking (200 rpm), after which a portion of each culture was transferred into fresh medium containing the same treatment. This serial transfer procedure was repeated daily for ten consecutive days. Independent replicate populations of *Escherichia coli* C were established from isolated colonies of either the wild‐type ancestor (EC‐WT) or the phage‐resistant rfaP mutant ancestor (EC‐P‐Res). Each selective condition (3 total) therefore consisted of four independently evolved populations, for each of the two ancestors, resulting in 24 evolved populations.

Ampicillin was selected as a representative β‐lactam antibiotic because of its well‐characterized mechanism of action and widespread use in studies of bacterial adaptation. Gallium nitrate was selected because gallium disrupts bacterial iron metabolism and has emerged as a promising nontraditional antimicrobial agent with demonstrated activity against Gram‐negative bacteria, making it an ideal candidate for investigating evolutionary responses to combined antimicrobial stress.

A minimum inhibitory concentration (MIC) assay utilizing the ancestral *E. coli* strain C was performed to determine sublethal concentrations suitable for experimental evolution. Concentrations were selected to inhibit bacterial growth while allowing continued population propagation under selection. Based on the MIC assay, populations were evolved in 40 mg/L ampicillin, 1500 mg/L gallium nitrate [Ga(NO_3_)_3_], or a combination of both concentrations throughout the evolution experiment.

Control populations of both EC‐WT and EC‐P‐Res were maintained in LB medium without ampicillin or gallium nitrate under identical incubation and transfer conditions to distinguish treatment‐specific evolutionary responses from adaptation to laboratory culture conditions.

### 2.3 Phenotypic Assays

Each of the evolved bacteria was tested with a phage infectivity spot assay to determine phage resistance. Spot assays were performed following standard procedures (Carlson 2005). We plated lawns with 100 uL of each bacterial population at the evolution end point. We conducted spot assays by adding ~2000 virions of each phage in 2 uL droplets to each bacterial lawn and incubating at 37°C for 24 h (Carlson 2005). Clear zones indicating viral lysis of bacterial cells were scored as 2 for complete clearing (no turbidity); 1 for partial clearing (opaque or turbidity), and 0 for no clearing. All assays were conducted in triplicate on each plate and across triplicate plates.

Measurements of 24‐h growth in 40 mg/L of ampicillin or 1500 mg/L of Ga(NO_3_)_3_ were determined at 10 days of evolution. The growth fitness of each bacterial population was tested determining OD600 in a 96‐well plate.

#### 2.3.1 Genotypic Assays

DNA was extracted from bacterial cultures using the Qiagen DNeasy Ultraclean Microbial Kit (12224‐50) as per manufacturer instructions. DNA samples were quantified using a Nanodrop 2000 and the 260/280 ratios for each sample were ~1.8, as expected for double‐stranded DNA. Extracted DNA samples were sent to SeqCenter for genomic library preparation using the Illumina Nextera XT kit and samples were sequenced using the Illumina MiSeq sequencing platform. Sequence alignment and variant calling from the samples was achieved by use of the *breseq* 0.30.0 pipeline using the reference *E. coli* C strain accession number CP020543.1 (GenBank). The breseq pipeline uses three types of evidence to predict mutations: read alignments, missing coverage and new junctions, and any reads that indicate a difference between the sample and the reference genome that cannot be resolved to describe precise genetic changes are listed as “unassigned.” These unassigned reads are not described or interpreted here.

### 2.4 Statistical Analyses

Statistical analyses were conducted in R (R Core Team 2021). Statistical significance was assessed using α = 0.05 throughout all analyses. A one‐way ANOVA was used to determine the overall effects of antibiotic, resistance, and the interaction on the response variable, zone of inhibition. The emmeans package allows for post hoc comparisons between groups after fitting a model (Lenth 2019). These subsequent pairwise contrasts were conducted to compare the zone of inhibition of *E. coli* WT (control group) to each phage‐resistant *E. coli* mutant (treatment group) for each antibiotic. This set of comparisons can be requested via trt.vs.ctrl. A Dunnett's test was used to correct for multiple comparisons, which is the default multiple comparisons adjustment used by the emmeans package.

## 3 Results

### 3.1 Figures and Tables Are at the End of the Main Document for Reviewers

Experimental evolution under ampicillin, gallium nitrate, and combined selective pressures resulted in increased resistance across multiple treatment environments. Many evolved populations exhibited increased growth under selective conditions relative to their respective ancestors, indicating the evolution of resistance to one or more stressors.

**FIGURE 1:**
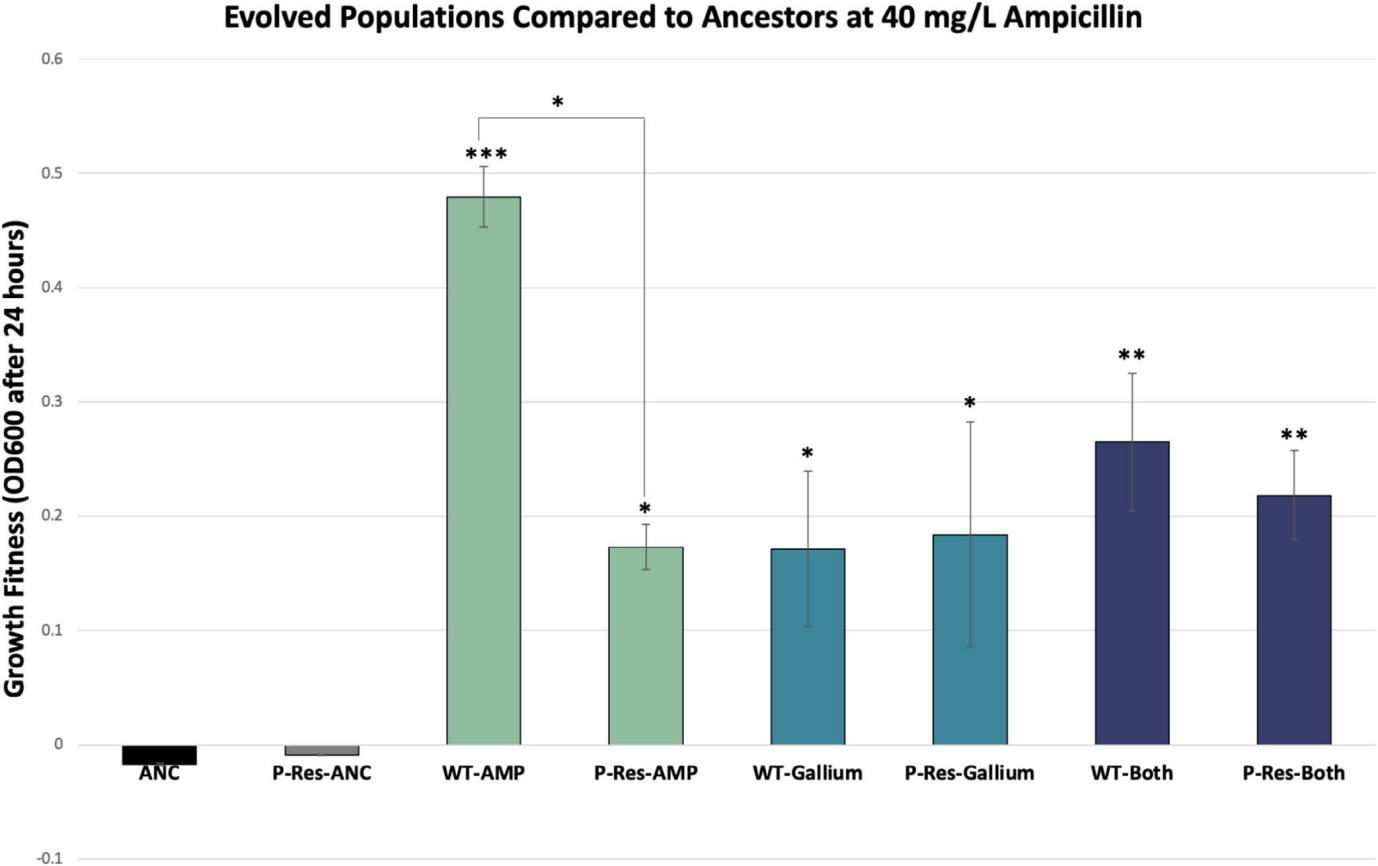
The growth fitness of evolved *E. coli* populations compared to ancestral strains under 40 mg/L ampicillin stress condition. The growth fitness of different *E. coli* strains were measured as 0D600 after 24 h in a 96‐well plate assay. All evolved populations exhibited significantly increased growth compared to the wild‐type ancestor (ANC) and phage‐resistant ancestor (P‐Res‐ANC). The WT‐AMP population showed the highest growth, significantly outgrowing the phage‐resistant counterpart (P‐Res‐AMP) (*p* < 0.05). No significant growth differences were observed between WT‐Gallium and P‐Res‐Galium or between WT‐Both and P‐Res‐Both. Error bars represent standard deviation across replicates. Asterisks indicate significance relative to the corresponding ancestral control: *p < 0.05, **p < 0.01, ***p < 0.001, **p < 0.0001; ns = not significant.

**FIGURE 2:**
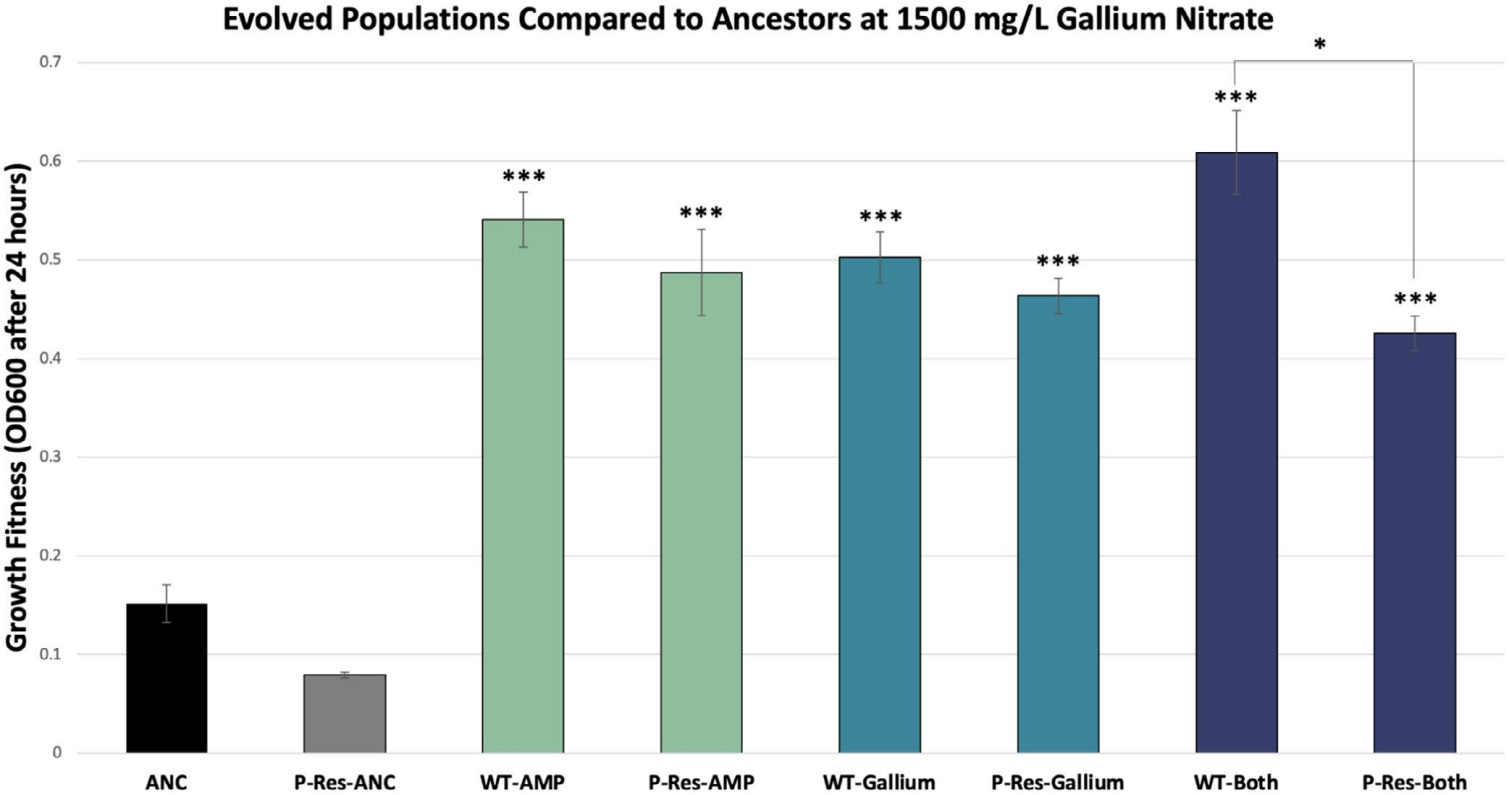
The growth fitness of evolved *E. coli* populations compared to ancestral strains under 1500 mg/L gallium nitrate stress condition. Growth fitness was measured as OD600 after 24 h in a 96‐well plate assay. All evolved populations showed significantly increased growth compared to the wild‐type ancestors (ANC) and the phage‐resistant ancestors (P‐Res‐ANC) (*p* < 0.001). No significant growth differences were observed between WT‐AMP and P‐Res‐AMP or between WT‐Gallium and P‐Res‐Gallium. However, a significant difference was observed between WT‐Both and P‐Res‐Both, with WT‐Both exhibiting growth (*p* < 0.05). Error bars represent standard deviation across replicates. Asterisks indicate significance relative to the corresponding ancestral control: *p < 0.05, **p < 0.01, ***p < 0.001, **p < 0.0001; ns = not significant.

Growth fitness of evolved populations under ampicillin and gallium nitrate selection is shown in Figures 1 and 2. Figures 1 and 2 summarize the mean growth fitness of replicate populations evolved under each selective condition.

### 3.2 Ampicillin Environment (40 mg/L)

All populations, regardless of their selection condition, demonstrated significantly increased growth compared to both the wild‐type ancestor (ANC) and the phage‐resistant ancestor (P‐Res‐ANC) (Figure 1). The WT‐AMP population exhibited the highest fitness under ampicillin exposure (*p* < 0.001), followed by modest but significant increases in P‐Res‐AMP, WT‐Gallium, P‐Res‐Gallium, WT‐Both, and P‐Res‐Both populations (*p* < 0.05 to *p* < 0.01). Notably, WT‐AMP showed significantly greater growth than P‐Res‐AMP (*p* < 0.05), indicating that prior phage resistance may constrain adaptive potential under ampicillin selection. There were no significant differences in growth between WT‐Gallium and P‐Res‐Gallium, nor between WT‐Both and P‐Res‐Both, suggesting that the phage resistance background did not impact growth fitness under ampicillin when the selection history involved gallium or both stressors.

### 3.3 Gallium Nitrate Environment (1500 mg/L)

Under gallium stress (Figure 2), all evolved populations again displayed significantly higher growth fitness than their ancestral counterparts (*p* < 0.001). Interestingly, populations evolved under ampicillin selection alone (WT‐AMP and P‐Res‐AMP) also demonstrated robust growth when exposed to gallium, suggesting potential cross‐resistance or generalized stress adaptations. There were no significant differences in growth between WT‐AMP and P‐Res‐AMP, nor between WT‐Gallium and P‐Res‐Gallium, indicating that the phage resistance background did not substantially influence growth fitness under gallium stress for these selection histories. However, a significant difference was observed between WT‐Both and P‐Res‐Both (*p* < 0.05), with WT‐Both showing greater fitness, suggesting that phage resistance may have limited the adaptive benefit under dual‐stressor conditions.

### 3.4 Genomic Mutations and Evolved Populations

We summarized the mutations detected at or above a frequency threshold of 0.25 in each population. Tables 1 and 2 summarize mutations detected at frequencies ≥ 0.25 in each evolved population. Reported mutations include nucleotide substitutions, amino acid substitutions, and the corresponding gene affected.

**TABLE 1:** High‐frequency mutations identified in evolved *E. coli* populations compared to the wild‐type ancestor.

Bacterial population	Selective condition	Nucleotide position	Nucleotide substitutions	AA substitutions	Gene	Gene function	Allele frequency
EC‐WT	None	—	—	—	—	—	—
EC‐WT‐AMP	Ampicillin	4,136,844	C → T	T118I	frdD	Fumarate reductase subunit D	1.00
	Ampicillin	4,322,474	C → T	E69K	rpoB	DNA‐directed RNA polymerase subunit beta’	1.00
	Ampicillin	445,243	C → T	P486L	fusA	Elongation factor G	1.00
EC‐WT‐GALL	Gallium nitrate	3,039,509	C → T	A17V	yhdW	Amino acid ABC transporter substrate‐binding protein	0.31
	Gallium nitrate	3,923,032	G → A	R163H		DNA‐binding response regulator	0.26
	Gallium nitrate	445,831	T → G	M682R	fusA	Elongation factor G	0.91
EC‐WT‐BOTH	Amp + gallium	4,136,844	C → T	T118I	frdD	Fumarate reductase subunit D	1.00
	Amp + gallium	4,404,514	G → A	E91K	rcsC	Two‐component sensor histidine kinase	1.00

**TABLE 2:** High‐frequency mutations identified in evolved *E. coli* populations compared to the phage‐resistant ancestor.

Bacterial population	Selective condition	Nucleotide position	Nucleotide substitutions	AA substitutions	Gene	Gene function	Allele frequency
EC‐P‐Res	None	75,889	A → T	H160L	rfaP	Lipopolysaccharide core heptose(I) kinase	1.00
EC‐P‐Res‐AMP	Ampicillin	75,889	A → T	H160L	rfaP	Lipopolysaccharide core heptose(I) kinase	1.00
	Ampicillin	368,706	G → A	A10A	YiaN/M	2,3‐diketo‐l‐gulonate TRAP transporter permease	0.93
	Ampicillin	2,900,850	Δ39 bp	coding	phoE	Phosphoporin PhoE	0.76
EC‐P‐Res‐GALL	Gallium nitrate	75,889	A → T	H160L	rfaP	Lipopolysaccharide core heptose(I) kinase	1.00
	Gallium nitrate	2,900,361	A → C	intergenic	phoE	Asparagine‐tRNA ligase/phosphoporin PhoE	0.5
	Gallium nitrate	443,864	T → G	T26T	fusA	Elongation factor G	0.25
	Gallium nitrate	4,331,617	T → G	E156D	tufA	Translation elongation factor EF‐Tu 2	0.44
	Gallium nitrate	1,586,505	A → C	D127A	phoE	Phosphoporin PhoE	0.72
	Gallium nitrate	3,701,377	C → T	D127A	nlpE	Copper homeostasis/adhesion lipoprotein NlpE	0.29
EC‐P‐Res‐BOTH	Amp + gallium	75,889	A → T	H160L	rfaP	Lipopolysaccharide core heptose(I) kinase	1.00
	Amp + gallium	1,970,219	T → G	L133R	mepM	Murein DD‐endopeptidase MepM	1.00
	Amp + gallium	3,826,984	T → C	N255S	ftsl	Peptidoglycan glycosyltransferase FtsI	0.64

Each evolved condition produced distinct sets of mutations, with some overlap across populations. For example, mutations were observed in *rpoB* (which encodes DNA‐dependent RNA polymerase, RNAP), *fusA* (which encodes elongation G), and *rcsC* (which encodes a sensor histidine kinase) among different treatment groups (Tables 1 and 2). The ancestor population with phage resistant mutation in the *rfaP* gene (also called *waaP*, which encodes a kinase involved in the biosynthesis of the core oligosaccharide region of lipopolysaccharide) resulted in all evolved populations maintaining this phage resistant mutation throughout the evolution experiments, even though phage resistance was not being selected for (Table 2). We confirmed the maintenance of phage resistance with spot assays to detect phage lysis, and observed that all evolved phage‐resistant populations produced no clear, lytic zones when infected with ID8 Microvirid bacteriophage. Populations evolved under the same selective environment frequently acquired mutations in different loci, though some genes were repeatedly affected across replicates or conditions (Tables 1 and 2). Both coding region mutations and regulatory region mutations were included in the analysis.

## 4 Conclusions and Implications

Our initial hypothesis predicted that combinatorial selective pressures—phage resistance background, ampicillin exposure, gallium nitrate exposure, and their combination—would constrain adaptation and limit the emergence of multi‐resistant phenotypes (McGee et al. 2023; Choi et al. 2020; Chaudhry et al. 2017). Instead, our results demonstrate that *Escherichia coli* populations readily evolved resistance across multiple stressors, frequently acquiring cross‐resistance even when exposed to only a single selective condition. These findings are consistent with previous studies demonstrating the remarkable adaptive capacity of bacterial populations under antimicrobial selection (Andersson 2003; Andersson and Hughes 2010). These findings suggest that evolutionary constraints such as fitness trade‐offs, pleiotropy, or epistatic interactions did not strongly limit adaptive trajectories over the ten‐day experimental period (Stearns 2010; McGee et al. 2023). Rather than trapping populations in fitness valleys, the imposed environments appear to have promoted access to generalized or overlapping resistance mechanisms (Steinberg and Ostermeier 2016a; Wright 1932).

To our surprise, all evolved populations, both wild‐type and phage resistant backgrounds, displayed significantly improved growth relative to their ancestors under both ampicillin and gallium stress. This outcome contrasts with previous work demonstrating more specialized adaptation to gallium exposure (Graves et al. 2019; Choi et al. 2020). Notably, populations selected solely under ampicillin conditions exhibited enhanced growth under gallium exposure, and vice versa. This pattern indicates cross‐resistance or the evolution of generalized stress‐response adaptations (Miller and Arias 2024; Lin et al. 2022). Such outcomes are consistent with the possibility that resistance mechanisms may confer protection across distinct stressors (Kortright et al. 2019; Principi et al. 2019).

These results are in contrast to the gallium resistance evolved in Graves et al. (2019) which utilized *E. coli* K‐12 MG1655. Gallium resistance here involved genes such as *fecA*, *fecB*, and *fecE* that encode proteins that were directly involved in the uptake of iron (for which gallium is an analog). Each of the five gallium‐resistant replicates had at least one *fecA* mutation and some also had mutations in *fecB* and *fecE*. These populations were also cross‐resistant against excess iron‐III and ionic silver (Ag+), but not against any antibiotics. These results indicated that the Graves et al. (2019) populations evolved a specific anti‐gallium mechanism that was neutral to, and in some cases (rifampicin) antagonistic against antibiotic resistance. This is an illustration of how historical contingency (in the form of fitness epistasis) shapes adaptive potential.

Another example of this was illustrated by the presence of a pre‐existing phage‐resistance mutation, *rfaP*, which also shaped the adaptive potential of the *E. coli* strain used in this experiment (McGee et al. 2023). All phage‐resistant lineages retained the *rfaP* mutation throughout the experiment, even in the absence of phage selection, and spot assays confirmed sustained resistance to ID8 Microviridae bacteriophage. This stability suggests that the phage‐resistance mutation either carried minimal fitness cost under our experimental conditions or that compensatory mutations may have mitigated any initial cost (Seed et al. 2012; McGee et al. 2023).

However, subtle constraints were observed. Under ampicillin selection alone, WT‐AMP populations achieved significantly higher growth than P‐Res‐AMP populations containing the *rfaP* mutation, indicating that prior phage resistance may have slightly reduced adaptive potential in this environment (McGee et al. 2023). Similarly, under dual‐stressor conditions, WT‐Both outperformed P‐Res‐Both populations under gallium stress. These findings suggest that epistatic interactions between the ancestral phage‐resistance mutation, *rfaP*, and newly acquired resistance mutations may have influenced evolutionary trajectories (Burmeister et al. 2020). While these constraints were not strong enough to prevent multi‐resistance evolution, they demonstrate that historical genetic background can shape the magnitude and direction of adaptation (McGee et al. 2023; Stearns 2010).

Whole‐genome sequencing revealed unique mutational pathways across replicate populations, with some convergence at the gene level. Mutations in genes such as *fusA*, phoE (which encodes an outer membrane porin), and *frdD* (which encodes fumarate reductase subunit D) were detected across multiple treatment groups. We also detected some known mutations in genes that have been reported from other studies exploring antibiotic resistance, such *as rpoB* and *rscC*. Mutations in *rpoB* and *fusA*, which encode components involved in transcription and translation, are frequently associated with global regulatory changes and antibiotic resistance (Andersson 2003; Andersson and Hughes 2010). Also, mutations in rpo's are associated with general improvements in fitness in a variety of experimental evolution studies in *E. coli* (Barrick et al. 2010; Graves Jr et al. 2015; González‐González et al. 2024). We observed mutations in *rpoB* in populations of wild‐type background when exposed to ampicillin treatment. Mutations in *rpoB* have been shown to increase osmotic stress resistance. These mutations, commonly found in *E. coli* , allow resistance to rifampin and can also be present when the bacteria rapidly evolve resistance to gallium (III) nitrate (Jeje et al. 2023). Alterations in *phoE*, an outer membrane porin, may influence membrane permeability and thereby affect susceptibility to both antibiotics and metal ions (Kaneko et al. 2007; Graves et al. 2019). We detected mutations in the *phoE* gene in populations exposed to ampicillin and gallium when the *rfaP* phage resistant background was present, but not when exposed to alternating treatment of ampicillin and gallium.

Mutations in *fusA* were detected in populations under ampicillin and gallium nitrate conditions, and in both the wild‐type and phage‐resistance backgrounds. The *fusA* gene is important in translational elongation, and particularly susceptible to oxidative stress in *E. coli* (Nagano et al. 2012). The elongation factor *fusA* has been detected in experiments identifying aminoglycoside resistance (Lázár et al. 2013). Other previous works suggest that resistance conferred by *fusA* mutations in other bacterial species can enhance sensitivity to other classes of antimicrobial agents (Macvanin and Hughes 2005), as we observe here in the presence of ampicillin.

Other notable mutations include those detected in the *frdD* gene, which have been found to influence transcription levels of ampC, which is a beta‐lactam gene necessary for ampicillin resistance (Teichmann et al. 2025). Another interesting finding was a mutation in the *rscC* gene which is a sensory histidine kinase and part of a two‐component response phospho‐relay system that evolved in the wild‐type background when exposed to both ampicillin and gallium nitrate treatment. Mutations in the *rcsC* system have been found to increase resistance to β‐lactam antibiotics by activating the RcsB response regulator. This can lead to the initiation of gene transcription that increases resistance and survival, including those involved in capsule synthesis and cell membrane integrity (Laubacher and Ades 2008).

Importantly, many detected mutations remained at frequencies near the reporting threshold (≥ 0.25), suggesting that adaptive sweeps were incomplete at the conclusion of the experiment. This indicates ongoing clonal interference and competition among beneficial mutations. Additional transfers would likely clarify which mutations ultimately dominate and whether certain pathways consistently outcompete others. The presence of multiple low‐frequency mutations further supports the idea that several accessible adaptive routes exist under these selective regimes and is consistent with what we observe concerning clonal interference in much longer‐term experimental evolution studies (McGee et al. 2023; Lin et al. 2022; Rose et al. 2026).

Our findings provide limited evidence for fitness trade‐offs between resistance to phage, antibiotics, and gallium nitrate. Instead, pleiotropic mutations may have conferred simultaneous benefits across stressors (Choi et al. 2020; Stearns 2010). While heavy metal resistance has been proposed to impose metabolic costs or create antagonistic trade‐offs (Graves Jr et al. 2015; Graves et al. 2019), our experimental evolution employed a concentration intended to impose strong but non‐lethal selective pressure that permitted continued bacterial growth and adaptation over successive transfers. Our results suggest that, at least in the short term, *E. coli* populations can overcome these constraints. From an evolutionary landscape perspective, combinatorial treatments did not consistently create adaptive valleys deep enough to prevent resistance evolution (Steinberg and Ostermeier 2016a; Wright 1932). Instead, environmental complexity may have reshaped the landscape in a way that favored mutations with broad protective effects. This observation has important implications for antimicrobial strategy design: simply layering selective pressures may not be sufficient to prevent the evolution of multi‐drug resistance (Kortright et al. 2019; Lin et al. 2022).

## Author Contributions

**Lindsey W. McGee:** conceptualization (lead), data curation (lead), formal analysis (lead), funding acquisition (equal), project administration (lead), supervision (lead), writing – review and editing (equal). **Noah K. Radtke:** data curation (supporting), investigation (supporting), writing – original draft (equal). **Gretchen S. Taylor:** investigation (equal), writing – original draft (equal), writing – review and editing (equal). **Joseph L. Graves Jr.:** conceptualization (equal), formal analysis (equal), writing – review and editing (equal). **Liesl Jeffers‐Francis:** conceptualization (equal), funding acquisition (equal), project administration (equal), writing – review and editing (equal).

## Funding

This work was supported by Stephensen Fund ‐ Earlham College and Division of Integrative Organismal Systems (2132240).

## Conflicts of Interest

The authors declare no conflicts of interest.

## Data Availability

Any data not made available directly in the manuscript are available at Science Data Bank: https://www.scidb.cn/en/s/rA3UBr.

## References

[ece374239-bib-0001] Andersson, D. I. 2003. “Persistence of Antibiotic Resistant Bacteria.” Current Opinion in Microbiology 6: 452–456. 14572536 10.1016/j.mib.2003.09.001

[ece374239-bib-0002] Andersson, D. I. , and D. Hughes . 2010. “Antibiotic Resistance and Its Cost: Is It Possible to Reverse Resistance?” Nature Reviews. Microbiology 8: 260–271. 20208551 10.1038/nrmicro2319

[ece374239-bib-0003] Barrick, J. E. , M. R. Kauth , C. C. Strelioff , and R. E. Lenski . 2010. “ *Escherichia coli* rpoB Mutants Have Increased Evolvability in Proportion to Their Fitness Defects.” Molecular Biology and Evolution 27, no. 6: 1338–1347. 10.1093/molbev/msq024. 20106907 PMC2872623

[ece374239-bib-0004] Bateman, A. , M. J. Martin , S. Orchard , et al. 2021. “UniProt: The Universal Protein Knowledgebase in 2021.” Nucleic Acids Research 49: D480–D489. 10.1093/nar/gkaa1100. 33237286 PMC7778908

[ece374239-bib-0005] Bertani, G. 1951. “Studies on Lysogenesis. I. The Mode of Phage Liberation by Lysogenic *Escherichia coli* .” Journal of Bacteriology 62, no. 3: 293–300. 14888646 10.1128/jb.62.3.293-300.1951 PMC386127

[ece374239-bib-0043] Burmeister, A. R. , A. Fortier , C. Roush , et al. 2020. “Pleiotropy Complicates a Trade‐Off Between Phage Resistance and Antibiotic Resistance.” Proceedings of the National Academy of Sciences of the United States of America 117, no. 41: 25607–25616. 10.1073/pnas.1919888117 PMC7260982 32424102

[ece374239-bib-0041] Carlson, K. 2005. “Working With Bacteriophages: Common Techniques and Protocols.” In Bacteriophages: Biology and Applications, edited by E. Kutter and A. Sulakvelidze , 437–456. CRC Press.

[ece374239-bib-0006] Chaudhry, W. N. , J. Concepcion‐Acevedo , T. Park , S. Andleeb , J. J. Bull , and B. R. Levin . 2017. “Synergy and Order Effects of Antibiotics and Phages in Killing *Pseudomonas aeruginosa* Biofilms.” PLoS One 12: e0168615. 10.1371/journal.pone.0168615. 28076361 PMC5226664

[ece374239-bib-0007] Choi, H. , Z. Yang , J. C. Weisshaar , and S. Diamond . 2020. “Gallium Resistance and Adaptation in *Escherichia coli* .” Journal of Bacteriology 202: e00064‐20. 10.1128/JB.00064-20. 32205461 PMC7221260

[ece374239-bib-0008] Coulter, L. B. , R. J. McLean , R. E. Rohde , and G. M. Aron . 2014. “Effect of Bacteriophage Infection in Combination With Tobramycin on the Emergence of Resistance in *Escherichia coli* and *Pseudomonas aeruginosa* Biofilms.” Viruses 6: 3778–3786. 10.3390/v6103778. 25285538 PMC4213561

[ece374239-bib-0009] De Oliveira, D. M. , B. M. Forde , T. J. Kidd , et al. 2020. “Antimicrobial Resistance in ESKAPE Pathogens.” Clinical Microbiology Reviews 33: e00181‐19. 10.1128/CMR.00181-19. 32404435 PMC7227449

[ece374239-bib-0010] González‐González, A. , T. N. Batarseh , A. Rodríguez‐Verdugo , and B. S. Gaut . 2024. “Patterns of Fitness and Gene Expression Epistasis Generated by Beneficial Mutations in the Rho and rpoB Genes of *Escherichia coli* During High‐Temperature Adaptation.” Molecular Biology and Evolution 41, no. 9: msae187. 10.1093/molbev/msae187. 39235107 PMC11414761

[ece374239-bib-0011] Graves, J. L. , A. J. Ewunkem , J. Ward , et al. 2019. “Experimental Evolution of Gallium Resistance in *Escherichia coli* .” Evolutionary Medicine and Public Health 2019: 169–180. 10.1093/emph/eoz017. PMC6928379 31890209

[ece374239-bib-0012] Graves, J. L., Jr. , M. Tajkarimi , Q. Cunningham , et al. 2015. “Rapid Evolution of Silver Nanoparticle Resistance in *Escherichia coli* .” Frontiers in Genetics 6: 42. 10.3389/fgene.2015.00042. 25741363 PMC4330922

[ece374239-bib-0013] Hobman, J. L. , and L. C. Crossman . 2015. “Bacterial Antimicrobial Metal Ion Resistance.” Journal of Medical Microbiology 64: 471–497. 10.1099/jmm.0.000032. 25418738

[ece374239-bib-0014] Hyman, P. , and S. T. Abedon . 2010. “Bacteriophage Host Range and Bacterial Resistance.” Advances in Applied Microbiology 70: 217–248. 10.1016/S0065-2164(10)70007-1. 20359459

[ece374239-bib-0015] Jeje, O. , A. J. Ewunkem , L. K. Jeffers‐Francis , and J. L. Graves Jr. 2023. “Serving Two Masters: Effect of *Escherichia coli* Dual Resistance on Antibiotic Susceptibility.” Antibiotics 12, no. 3: 603. 10.3390/antibiotics12030603. 36978471 PMC10044975

[ece374239-bib-0016] Kaneko, Y. , M. Thoendel , O. Olakanmi , B. E. Britigan , and P. K. Singh . 2007. “The Transition Metal Gallium Disrupts *Pseudomonas aeruginosa* Iron Metabolism and Has Antimicrobial and Antibiofilm Activity.” Journal of Clinical Investigation 117: 877–888. 10.1172/JCI30187. 17364024 PMC1810576

[ece374239-bib-0017] Kortright, K. E. , B. K. Chan , J. L. Koff , and P. E. Turner . 2019. “Phage Therapy: A Renewed Approach to Combat Antibiotic‐Resistant Bacteria.” Cell Host & Microbe 25: 219–232. 10.1016/j.chom.2019.01.014. 30763536

[ece374239-bib-0018] Laubacher, M. E. , and S. E. Ades . 2008. “The Rcs Phosphorelay Is a Cell Envelope Stress Response Activated by Peptidoglycan Stress and Contributes to Intrinsic Antibiotic Resistance.” Journal of Bacteriology 190, no. 6: 2065–2074. 10.1128/JB.01740-07. 18192383 PMC2258881

[ece374239-bib-0019] Lázár, V. , G. Pal Singh , R. Spohn , et al. 2013. “Bacterial Evolution of Antibiotic Hypersensitivity.” Molecular Systems Biology 9: 700. 10.1038/msb.2013.57. 24169403 PMC3817406

[ece374239-bib-0020] Lemire, J. A. , J. J. Harrison , and R. J. Turner . 2013. “Antimicrobial Activity of Metals: Mechanisms, Molecular Targets and Applications.” Nature Reviews. Microbiology 11: 371–384. 10.1038/nrmicro3028. 23669886

[ece374239-bib-0042] Lenth, R. V. 2019. “emmeans: Estimated Marginal Means, aka Least‐Squares Means.” R Package Version 1.4.1. https://CRAN.R‐project.org/package=emmeans.

[ece374239-bib-0021] Lin, D. M. , B. Koskella , and H. C. Lin . 2022. “Phage Therapy: An Alternative to Antibiotics in the Age of Multidrug Resistance.” Annual Review of Microbiology 76: 459–477. 10.1146/annurev-micro-030422-051038.

[ece374239-bib-0022] Loc‐Carrillo, C. , and S. T. Abedon . 2011. “Pros and Cons of Phage Therapy.” Bacteriophage 1: 111–114. 10.4161/bact.1.2.14590. 22334867 PMC3278648

[ece374239-bib-0023] Macvanin, M. , and D. Hughes . 2005. “Hyper‐Susceptibility of a Fusidic Acid‐Resistant Mutant of Salmonella to Different Classes of Antibiotics.” FEMS Microbiology Letters 247, no. 2: 215–220. 10.1016/j.femsle.2005.05.007. 15935566

[ece374239-bib-0024] McGee, L. W. , Y. Barhoush , R. Shima , and M. Hennessy . 2023. “Phage‐Resistant Mutations Impact Bacteria Susceptibility to Future Phage Infections and Antibiotic Response.” Ecology and Evolution 13: e9712. 10.1002/ece3.9712. 36620417 PMC9817185

[ece374239-bib-0025] Miller, W. R. , and C. A. Arias . 2024. “ESKAPE Pathogens: Antimicrobial Resistance, Epidemiology, Clinical Impact and Therapeutics.” Nature Reviews Microbiology 22: 598–616. 10.1038/s41579-024-00704-2. 38831030 PMC13147291

[ece374239-bib-0026] Nagano, T. , K. Kojima , T. Hisabori , et al. 2012. “Elongation Factor G Is a Critical Target During Oxidative Damage to the Translation System of *Escherichia coli* .” Journal of Biological Chemistry 287, no. 34: 28697–28704. 10.1074/jbc.M112.378067. 22773838 PMC3436518

[ece374239-bib-0027] Principi, N. , E. Silvestri , and S. Esposito . 2019. “Advantages and Limitations of Bacteriophages for the Treatment of Bacterial Infections.” Frontiers in Pharmacology 10: 513. 10.3389/fphar.2019.00513. 31139086 PMC6517696

[ece374239-bib-0028] R Core Team . 2021. R: A Language and Environment for Statistical Computing. R Foundation for Statistical Computing.

[ece374239-bib-0029] Rokyta, D. R. , Z. Abdo , and H. A. Wichman . 2009. “The Genetics of Adaptation for Eight Microvirid Bacteriophages.” Journal of Molecular Evolution 69, no. 3: 229–239. 19693424 10.1007/s00239-009-9267-9 PMC2746890

[ece374239-bib-0030] Rose, M. R. , R. Lenski , M. Matos , and J. L. Graves . 2026. A Primer for Experimental Evolution. World Scientific Publishers.

[ece374239-bib-0031] Ryan, E. M. , M. Y. Alkawareek , R. F. Donnelly , and B. F. Gilmore . 2012. “Synergistic Phage‐Antibiotic Combinations for the Control of *Escherichia coli* Biofilms In Vitro.” FEMS Immunology and Medical Microbiology 65: 395–398. 10.1111/j.1574-695X.2012.00960.x. 22524448

[ece374239-bib-0032] Sambrook, J. , and D. W. Russell . 2001. Molecular Cloning: A Laboratory Manual. 3rd ed. Cold Spring Harbor Laboratory Press.

[ece374239-bib-0033] Seed, K. D. , S. M. Faruque , J. J. Mekalanos , S. B. Calderwood , F. Qadri , and A. Camilli . 2012. “Phase Variable O Antigen Biosynthetic Genes Control Expression of the Major Protective Antigen and Bacteriophage Receptor in *Vibrio cholerae* O1.” PLoS Pathogens 8: e1002917. 10.1371/journal.ppat.1002917. 23028317 PMC3441752

[ece374239-bib-0034] Stearns, F. W. 2010. “One Hundred Years of Pleiotropy: A Retrospective.” Genetics 186: 767–773. 10.1534/genetics.110.122549. 21062962 PMC2975297

[ece374239-bib-0040] Steinberg, B. E. , and M. Ostermeier . 2016b. “Environmental Oscillations and Negative Selection Promote Crossing of Fitness Valleys.” Journal of Molecular Biology 428, no. 15: 3065–3078.

[ece374239-bib-0035] Steinberg, B. , and M. Ostermeier . 2016a. “Environmental Changes Bridge Evolutionary Valleys.” Science Advances 2: e1500921. 10.1126/sciadv.1500921. 26844293 PMC4737206

[ece374239-bib-0036] Tagliaferri, T. L. , M. Jansen , and H. P. Horz . 2019. “Fighting Pathogenic Bacteria on Two Fronts: Phages and Antibiotics as Combined Strategy.” Frontiers in Cellular and Infection Microbiology 9: 22. 10.3389/fcimb.2019.00022. 30834237 PMC6387922

[ece374239-bib-0037] Teichmann, L. , M. Wenne , S. Luitwieler , G. Dugar , J. Bengtsson‐Palme , and B. Ter Kuile . 2025. “Genetic Adaptation to Amoxicillin in *Escherichia coli* : The Limited Role of dinB and katE.” PLoS One 20, no. 2: e0312223. 10.1371/journal.pone.0312223. 39970152 PMC11838884

[ece374239-bib-0039] World Health Organization . 2026. “Antimicrobial Resistance.” https://www.who.int/news‐room/fact‐sheets/detail/antimicrobial‐resistance.

[ece374239-bib-0038] Wright, S. 1932. “The Roles of Mutation, Inbreeding, Crossbreeding, and Selection in Evolution.” Proceedings of the Sixth International Congress on Genetics 1: 356–366.

